# Generating quantitative models describing the sequence specificity of biological processes with the stabilized matrix method

**DOI:** 10.1186/1471-2105-6-132

**Published:** 2005-05-31

**Authors:** Bjoern Peters, Alessandro Sette

**Affiliations:** 1 La Jolla Institute for Allergy and Immunology, 3030 Bunker Hill Street, Suite 326, San Diego, CA 92109, USA

## Abstract

**Background:**

Many processes in molecular biology involve the recognition of short sequences of nucleic-or amino acids, such as the binding of immunogenic peptides to major histocompatibility complex (MHC) molecules. From experimental data, a model of the sequence specificity of these processes can be constructed, such as a sequence motif, a scoring matrix or an artificial neural network. The purpose of these models is two-fold. First, they can provide a summary of experimental results, allowing for a deeper understanding of the mechanisms involved in sequence recognition. Second, such models can be used to predict the experimental outcome for yet untested sequences. In the past we reported the development of a method to generate such models called the Stabilized Matrix Method (SMM). This method has been successfully applied to predicting peptide binding to MHC molecules, peptide transport by the transporter associated with antigen presentation (TAP) and proteasomal cleavage of protein sequences.

**Results:**

Herein we report the implementation of the SMM algorithm as a publicly available software package. Specific features determining the type of problems the method is most appropriate for are discussed. Advantageous features of the package are: (1) the output generated is easy to interpret, (2) input and output are both quantitative, (3) specific computational strategies to handle experimental noise are built in, (4) the algorithm is designed to effectively handle bounded experimental data, (5) experimental data from randomized peptide libraries and conventional peptides can easily be combined, and (6) it is possible to incorporate pair interactions between positions of a sequence.

**Conclusion:**

Making the SMM method publicly available enables bioinformaticians and experimental biologists to easily access it, to compare its performance to other prediction methods, and to extend it to other applications.

## Background

Whenever experimental data is gathered to examine a process, researchers will try – implicitly or explicitly – to define a model describing the process. The purpose of building a model is to generalize the experimental data, allowing the prediction of new experimental outcomes, or to gain insights into the experimental process. In the biological sciences, models are often implicitly built and verbally formulated, such as "the proteasome preferably cleaves after hydrophobic amino acids". Such a model is easy to understand and often reflects all the knowledge that can be gathered from a few, difficult experiments. However, with the advent of high-throughput experiments to the biosciences, it has become feasible to generate more quantitative, mathematical models, based on large volumes of data. For the purpose of building a model, conducting experiments can be formally described as collecting pairs of experimental parameters x and experimental outcomes (measurements: y_meas_). Building a model is then equivalent to finding a function f(x) = y_pred _≈ y_meas_. Herein, we call the model function f a prediction tool, and the experimental observations used to generate it its training set T = (x, y_meas_).

Developing a prediction tool is a two step process. First, a general prediction method capable of generating specific tools has to be chosen, such as a certain type of neural network, classification tree, hidden markov model or regression function. This choice is to some degree determined by the experimental data available. However, only a few prediction methods are clearly unsuited for a certain type or amount of data, leaving several potentially appropriate methods to choose from. In practice, the personal experience of a scientist often determines which prediction method he applies, as learning to apply a new method is often more costly than the benefits of a slightly better model.

After a method is chosen, it is applied to the experimental data to generate a specific prediction tool. Several different terms are commonly used for this second step, such as 'supervised learning', 'fitting the model' or 'regression'. Each method has its own formalism describing how this is done, but essentially a method is capable of generating a certain class of functions, of which one is chosen, that minimizes the difference between measured and predicted experimental outcomes for the training set T.

Here we describe a computer program implementing the SMM prediction method, which can be applied to model the sequence specificity of quantifiable biological processes. In such experiments, the input parameter × corresponds to a sequences of amino-or nucleic acids, and the experimental outcome y_meas _is a real numbers measuring the process efficiency. The method utilizes training sets in which the examined sequences all have the same length.

The SMM method was previously applied successfully to predictions of MHC binding [1], TAP transport [2] and proteasomal cleavage [3]. However, its software implementation was not made publicly available, because it relied on a commercial library with restrictive licensing terms. Also, applying the method to a new problem required manual changes in the source code, which is impracticable for an external user. Both of these issues are addressed in the software implementation made available here. In addition, this manuscript describes two specific features that we have found to be effective in generating high quality models and which can easily be utilized in other prediction methods, namely handling of bounded data and combinatorial peptide libraries.

The SMM software is not meant for 'classical' sequence analysis, which can be roughly defined as aligning related sequences in order to identify conserved residues or in order to generate classifiers which can identify additional related sequences. Rather, the typical application is the characterization of a sequence recognition event, such as the sequence specific cleavage efficiency of a protease. This characterization does not assume any evolutionary relationship between different recognized sequences. In terms of scientific fields, that makes the SMM software aimed at applications in biochemistry or molecular biology.

## Implementation

### Code

The SMM algorithm is implemented in C++ code. Only standard libraries or freely available external libraries were used. The two external libraries are Tinyxml [4] to handle the XML input and output and the Gnu Scientific Library [5] for efficient vector and matrix operations. The source code has been compiled and tested using Visual C++ on a windows system and using g++ (1.5 or above) on a Debian and Suse Linux system. A windows executable is also available. The source code, documentation and examples are available as additional files 1 and 2 of this manuscript, and on the project homepage at .

On a standard 2.6 GHz Pentium 4 PC running windows XP, the creation of a prediction tool from a typical set of training data containing 300 8-mer peptides takes about 10 minutes.

### Input and output

**Figure 1:**
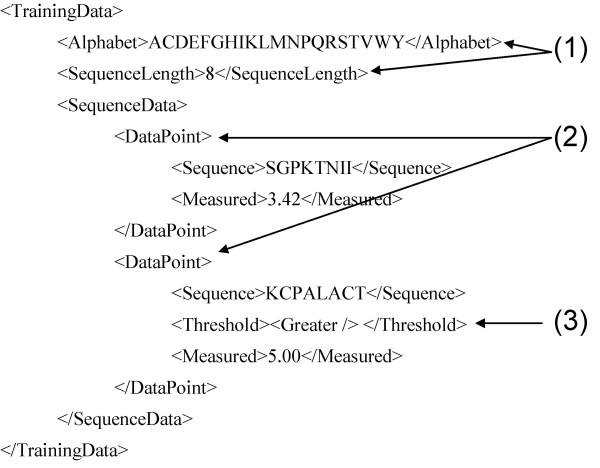
**Input training data. **The <TrainingData> element consists of a series of <DataPoints> (2). Each contains a sequence and a measurement value. The characters allowed in <Sequence> are specified in <Alphabet> (1), and the number of characters has to correspond to <SequenceLength> (1). In this example, <Alphabet> and <SequenceLength> specify 8-mer peptides in single letter amino acid code. Each measurement can optionally be associated with a threshold (3) that can either be <Greater> or <Lesser>, signaling that the measurement corresponds to an upper or lower boundary of measurable values.

The program expects an XML document as its standard input. A more sophisticated (graphical) user interface is not likely to be of great interest for the projected user community, most of which will probably call the executable file from a script or integrate the code into an existing program. The XML input document contains either training data to generate a new tool (Figure 1) or sequences for which a prediction should be made with a previously generated tool. The output of the program is again in XML format. For all types of input and output, XML schemas defining their exact structure are supplied. The schemas contain annotation for each input element, documenting their intended use. A simplified alternative input format exists for the most common application, namely the generation of a scoring matrix from a set of standard amino acid sequences. Several example input files are supplied with the program, which should make it easy for users to create similar input files with their own data.

### Core algorithm

An amino acid can be encoded as a binary vector of length 20, with zeros at all positions except the one coding for the specific amino acid. Extending this notation, a peptide of length N can be encoded as an N*20 binary vector. The same conversion can be made for nucleic residues, resulting in N*4 length vectors. Any fixed length sequence can be converted into a fixed length binary vector following similar rules. The SMM algorithm expects such a vector as the experimental input parameter 'x'.

**Figure 2:**
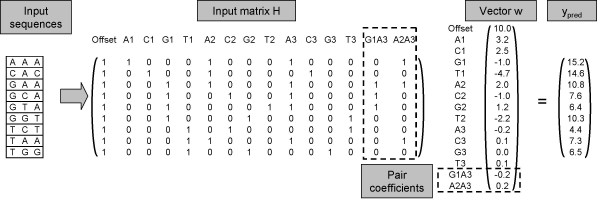
**Converting sequences into matrices. **Input sequences of three nucleic acids each are converted to rows of a matrix H. The first column of each row is set to 1, which serves as a constant offset added to each prediction. Columns A1 to T1 contain a binary representation of the first residue in the sequence, in which all columns are set to zero except the one corresponding to the residue. The same is repeated for the second and third residue in the sequence in columns A2 to T2 and A3 to T3. The two last columns G1A3 and A2A3 contain pair coefficients explained at the end of the results section. They are set to one if the two specified residues are present in the input sequence at the two specified positions and zero otherwise. Multiplying matrix H with the weight vector w results in a vector y_pred _of predicted values for the sequences. Rows A1 to T3 of vector w are commonly written as a 'scoring matrix' which quantifies the contribution of each possible residue at each position to the prediction. Rows G1A3 and A2A3 of vector w quantify the impact of the pair coefficients.

For a set of experiments, the vectors ^t^x (where ^t^x indicates the transposed vector x) can be stacked up resulting in a matrix H. An example for a set of nucleic sequences is shown in Figure 2. The matrix H is multiplied by a vector w which assigns a weight to each possible residue at each position in the sequence. This generates a vector of predictions y_pred_

H w = y_pred _    (1)

From a given training set of sequences encoded in H with measured values y_meas_, the 'correct' values for w can be found by minimizing the difference between the predicted values y_pred _and the measured values y_meas _according to a norm || ||. To suppress the effect of noise in the experimental data, a second term is added to the minimization:

||H w - y_meas_|| + ^t^w Λ w → minimum,    (2)

in which Λ is a positive scalar or a diagonal matrix with positive entries. To better understand the effect of the Λ term, consider first minimizing (2) with Λ set to zero. In this case, the optimal entries for the weight vector w minimize the difference between predicted (y_pred_) and measured (y_meas_) values. Minimizing (2) with a non-zero value for Λ results in a shift of the optimal entries in w towards values closer to zero, especially for entries in w that do not significantly decrease the distance between predicted and measured values. This technique, generally called regularization, suppresses the effect of noise in the measured data y_meas _on the entries in the weight vector w. Refer to [6] or [7] for a general introduction to regularization. If the || || norm is simply the sum squared error (or L2 norm), equation (2) can be solved analytically for any given value of Λ to:

w = (^t^HH + Λ)^-1 ^^t^H y_meas_   (3)

The optimal value for Λ can then be determined by cross validation: The experimental data points corresponding to rows of (H, y_meas_) are randomly separated into training sets T_i _= (H, y_meas_)_train i _and blind sets (H, y_meas_)_blind i_. For a given Λ, equation (3) is used to calculate w_i _for each training set T_i_. These w_i _can then be used to make predictions for their corresponding blind sets. Summing the distances for all blind predictions gives the cross validated distance Φ:

Φ(Λ) = Σ_i _|| H_blind i _w_i_(Λ) -y_blind i _||    (4)

Minimizing Φ as a function of Λ therefore corresponds to minimizing the cross validated distance by 'damping' the influence of coefficients in w which are susceptible to noise.

As the resulting optimal value for Λ and the corresponding w_i _are somewhat influenced by the split into training and blind sets, the same procedure is repeated several times with different splits, which is called bagging [8]. The final w is generated by averaging over all optimal w_i _generated in the cross validation and bagging repeats.

## Results and discussion

The following sections describe specific properties of the SMM method, and are meant to serve as a guideline when and how to apply it. Additional data validating the SMM algorithm and comparing it with other prediction methods can be found in previous publications [1-3].

### Linear model

If no pair coefficients are incorporated, the output vector w of the SMM method is a standard 'scoring matrix', which quantifies the contribution of each residue at each position in the input sequence to the prediction. Such a matrix is easy to interpret and analyze without requiring any additional software or expert knowledge of how the matrix was generated, which is especially important when communicating results to experimentalists. Several methods predicting peptide binding to MHC molecules take this approach, e.g. [9-12], and a comparative study showed that simple statistical methods to generate matrices can perform better than more complex artificial neural networks if the amount of data is limited [13].

Using such a linear model implicitly assumes that the influence of residues at each position in the sequence on the measured value can be considered independent and additive. This has to be a reasonable first approximation in order to successfully apply the SMM method, even if pair coefficients are incorporated. This is the main difference to general learning algorithms such as neural networks, which can in principle model any functional relationship between sequences and measurements.

### Quantitative data

The experimental measurements that serve as input to the SMM method and the predicted output are quantitative, not binary. For example, in the case of peptide binding to MHC molecules, IC50 values quantifying binding affinities are used, and not a classification into binding and non-binding peptides.

If different representations of the quantitative data are possible, such as either IC50 or log(IC50) values, a representation should be chosen in which the y_meas _values approximately follow a normal distribution. Otherwise the SMM predictions, which are sums of independent contributions and therefore roughly normally distributed themselves, will not be able to fit the experimental data well. In the case of binding affinities, this means log(IC50) values should be used, as IC50 values themselves are usually log normal distributed.

### Noisy data

**Figure 3:**
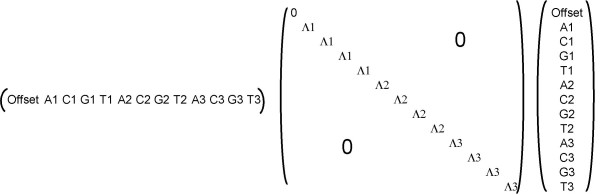
**Sequence position dependent regularization. **Example for the regularization term ^t^w Λ w in equation (2). The weight vector w corresponds to a scoring matrix for three nucleic acids as in Figure 2, but without pair coefficients. The diagonal matrix Λ has three different values Λ1, Λ2 and Λ3 effecting values in vector w corresponding to sequence positions 1, 2 and 3. There is no regularization penalty on the 'Offset' value.

Experimental measurements inevitably contain noise. This will cause problems when building models that take the measured values to be exact. Accordingly, the SMM method incorporates a regularization parameter Λ (equation 2), which corresponds to preferring a simpler solution with 'smooth' values for w to one that exactly reproduces observations. In the first SMM applications [1,2], Λ was a scalar, in which case this approach is called Ridge regression or zero-order regularization. Choosing a scalar value implicitly assumes that the level of signal to noise is roughly the same at each position in the input sequence. In the current version, Λ can also be chosen as sequence position dependent, which is sometimes called local Ridge regression. As shown for an example in Figure 3, this makes Λ into a diagonal matrix in which all Λ_i _values belonging to residues at the same sequence position are set to the same value. For a sequence of length N, there are N different Λ_i _values. For a number of training sets containing peptide binding data to MHC molecules, we compared the prediction performance achieved using a position specific matrix Λ_i _to a scalar Λ. The position specific regularization nearly always resulted in better predictions. The difference was especially large if the influence of different sequence positions varied greatly (data not shown).

### Bounded data

Any experimental technique generates measured values contained within a finite range. For example, in many biological experiments a "zero" measurement usually means that the actual value is below the experimental resolution, not that the actual value is 0. Similarly, very large values beyond the expected sensitivity limit are no longer quantitatively accurate. These data points at the upper or lower boundaries of the sensitivity range do not convey the same information as quantified values, but they still do contain information. In the case of MHC binding data available to us, approximately 20% of peptides fall in this category.

**Figure 4:**
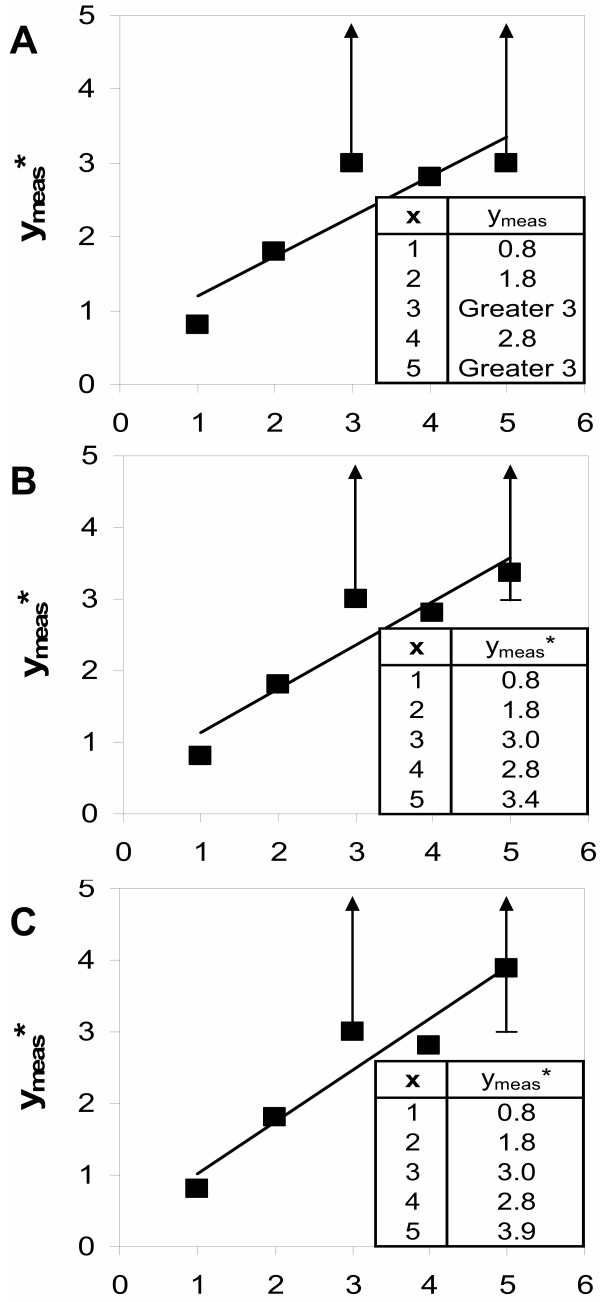
**Iterative model fitting using the L2_<> _norm. **In this example, the model is a linear function which is fitted to a set of paired values (x, y_meas_). For two of the x values (x = 3 and x = 5), the measured values are thresholds (Greater 3). Fitting a linear function to paired values according to the L2 norm corresponds to the standard linear regression. **A **depicts the model fit (straight line) to the measured values (black boxes), ignoring any thresholds. For x = 5, the model value y_pred _taken from the regression curve is 3.4, above the measured threshold value 3. Therefore, in the next iteration the y_meas_* value is set to the model value 3.4. **B **shows the new linear regression with the adjusted y_meas_* values. This procedure is repeated until the y_meas_* values no longer change (8 iterations, panel **C**).

The SMM method is, to the best of our knowledge, the only method designed to extract information from such boundary values. This is done by means of the novel L2_<> _norm, illustrated in Table 1. For example, if an experimental measurement corresponds to an upper boundary y_meas _> z, and the predicted value is greater than z, then the distance between y_meas _and y_pred _is zero. This norm has the useful property that any analytical solution according to the L2 norm can be converted into a solution according to the L2_<> _norm through an iterative process: First, all measurements including boundary values are treated as normal values, and the solution using the L2 norm is found. In a second step, for each y_pred_, y_meas _value pair for which the L2_<> _norm would be zero, the y_meas _value is set to its corresponding y_pred _value. For these y_meas_* values, the distance L2(y_pred_, y_meas_*) = L2_<> _(y_pred_, y_meas_). These y_meas_* values are then used to solve again according to the L2 norm. This process is repeated until the y_meas_* values no longer change, as illustrated in Figure 4.

### Randomized peptide library data

As stated before, the experimental data used as input for the SMM method consists of same length amino-or nucleic acid sequences associated with a quantitative measurement. When designing an experiment, the selection of sequences to test can introduce bias into the training data, for example by over-or under-representing residues at specific sequence positions. One way to avoid this is the use of randomized peptide libraries, also known as positional scanning combinatorial peptide libraries, which are mixtures of peptides of the same length. In a given library, all peptides have one residue in common at a fixed position and a random mixture of amino acids at all other positions. For example, the library XAXXXXXX contains 8-mer peptides with a common Alanine at position 2. Such libraries can be used to directly measure the influence of their common residue, by comparing their measured process efficiency to that of the completely randomized library XXXXXXXX. In the case of 8-mer peptides, 160 library experiments are sufficient to characterize the influence of each residue at each position. The results of such a complete scan can be summarized in a scoring matrix. This approach has been used successfully in many different experimental systems [14-17]

**Figure 5:**
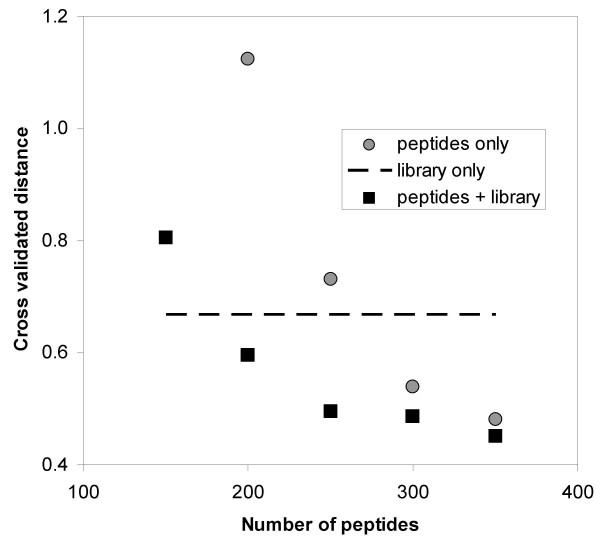
**Combining peptide and library data improves prediction quality. **A set of 449 9-mer peptides with measured affinities for TAP taken from [20] was split into 5 blind sets. For each of these blind sets, predictions where made from different size subsets of the remaining peptides. The x-axis depicts the number of peptides in these subsets used for generating predictions using either the peptides alone (circles) or in combination with data from a combinatorial peptide library (squares). The dashed line displays the prediction of the library alone, which was taken from [15]. The y-axis depicts the L2_<> _distance of the predictions for the combined 5 blind sets.

**Figure 6:**
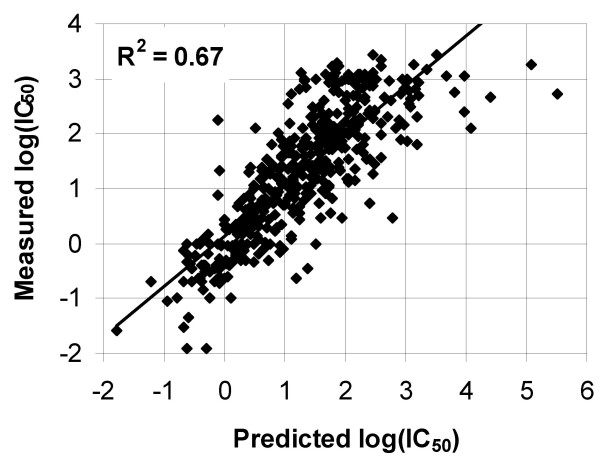
**Visualization of prediction quality. **Scatter plot of predicted vs. measured affinity for peptide binding to TAP. The depicted prediction corresponds to the data point in figure 5 with the lowest cross-validated distance, in which 350 peptides and the peptide library were used for training.

A novel feature of the SMM method is that it can combine data from these two sources. When the SMM algorithm is given experimental data from individual peptides and from a randomized library summarized in a scoring matrix mat_lib_, it simply subtracts the values predicted by mat_lib _from each individual peptide measurement. These y'_meas _= y_meas _- y_pred,lib _values are then used to generate a second scoring matrix mat'. The final SMM scoring matrix is simply the sum of the two: mat_combined _= mat' + mat_lib_. Figure 5 compares the performance of this combined approach to that of a prediction based on peptide or library experiments alone. If enough peptide data is present (roughly the same number as matrix parameters), the combined prediction is better than that of the library matrix alone. At all data points, the combined prediction is better than that using the peptides alone. Importantly, this simple strategy of subtracting library predictions can be used in combination with any prediction method, and is likely to generate similar results, as it effectively increases the training set size. To visualize the prediction quality associated with the distances reported in Figure 5, Figure 6 depicts a scatter plot of the predicted and measured binding affinity for individual peptides corresponding to the data point with the lowest distance in Figure 5.

### Introducing pair coefficients

Pair coefficients quantify the contribution of a pair of residues to the measured value that deviates from the sum of their individual contributions. The form of equation (1) remains unchanged if pair coefficients are introduced in the same binary notations as the individual coefficients. Figure 2 gives an example how a set of nucleic sequences is transformed into a matrix H, if two such pair coefficients are taken into account. Note that the number of possible pair coefficients is very large. For a sequence of three nucleic acids, there are already (3*4) * (2*4) / 2 = 48 pair coefficients. For a 9-mer peptide, (9*20) * (8 * 20) / 2 = 14400 pair coefficients exist. To the best of our knowledge, only the SMM method and the additive method [18] explicitly quantify the influence of pair coefficients.

Since most training sets contain only a few hundred measurements, determination of the exact values of all pair coefficients is not feasible. To overcome this difficulty, the SMM algorithm limits the number of pair coefficients to be determined. First, only coefficients for which sufficient training data exists are taken into account. As a rule of thumb, 5 sequences containing the same pair of residues at the same positions have to be present in the training set for a pair coefficient to be considered. In a second filtering step, only pair coefficients for which the information in the training set is reasonably consistent are retained. In the previous SMM version, this used to be determined by multiple fitting of pair coefficients and discarding those for which a sign change was observed. In the current version, a much faster approach is used. First, a scoring matrix is calculated for the training set without any pair coefficients. Then, for each pair coefficient the predicted and measured values for the sequences containing it are compared. Only if a large enough majority (>60%) of measured values are above or below the matrix based predictions is the pair coefficient retained. The remaining pair coefficients are determined in complete analogy to the scoring matrix itself, but with a scalar Λ value.

We tested the effect of incorporating pair coefficients on prediction quality compared to using a scoring matrix alone for a number of training sets containing peptide to MHC binding data. The pair coefficients showed a consistent positive contribution for large training sets, which comprise more measurements than 1.5 times the number of scoring matrix coefficients. However, the improvement is rather small, as reported before in [1]. This makes it reasonable to ignore pair coefficients if the simplicity of a scoring matrix is more valuable than a small improvement in prediction quality.

If higher order sequence interactions such as those described by pair coefficients are expected to be the dominant influence on experimental outcomes, other prediction methods may be better suited than the SMM method. For example, by choosing a different sequence representation than the binary vectors, the information in the training set can be generalized, thereby effectively reducing the degrees of freedom in the input parameters [19]. This allows applying general higher order learning algorithms such as artificial neural networks even with limited input data.

## Conclusion

The SMM method generates quantitative models of the sequence specificity of biological processes, which in turn can be used to understand and predict these processes. It has previously been shown to perform very well compared to other prediction methods and tools for three specific types of experimental data [1-3]. However, it is difficult to generalize a comparison between different methods, due to two main problems. First, the training data sets utilized in different studies are often not available, so that when comparing tools generated by different methods it is often unclear when good performance is due to a superior method or a better (larger) set of training data. Second, generating tools from the same training set can be difficult, because publications that make the tools available, often only describe the basic principle of the method used.

To overcome this second obstacle, we herein presented a computer program implementing the SMM method. Significant effort was devoted to ensuring that the program is robust, documented, cross platform compatible and generates reasonable output without requiring additional parameters. Also, any commercial libraries previously utilized were removed to allow free distribution of the code. This will permit any interested user to apply the SMM method with reasonable effort, allowing for the most important validation: application to scientific practice.

Finally, we believe that two strategies demonstrated in this manuscript will be valuable in combination with other prediction methods as well. First, our strategy for the inclusion of experimental data gathered with randomized peptide libraries can be directly transferred to any prediction method. When other experimental data is limited and data from a combinatorial library is available, this should always have a positive effect on prediction quality. Secondly, the L2_<> _norm can be applied as an error function for other prediction methods. This will increase the amount of training data effectively available to prediction methods requiring quantitative input, by enabling them to handle experimental boundary values.

## Availability and requirements

Project name: smm

Project home page: 

Operating system(s): Platform independent

Programming language: C++

Other requirements: The Gnu Scientific Library (GSL) has to be installed

License: None for the smm code, but GSL requires the GNU GPL license

Any restrictions to use by non-academics: None

## Authors' contributions

BP conceived of this study and implemented the software, AS participated in the design of the software and helped to draft the manuscript. All authors read and approved the final manuscript.

## Supplementary Material

Additional File 1 A .zip file containing the SMM source code, documentation and examples for windows systems. Click here for file

Additional File 2 A .tar.gz file containing the SMM source code, documentation and examples for linux systems. Click here for file

## Figures and Tables

**Table 1:** The L2_<> _norm

**Measurement**	**L2_<> _distance**	
	
	**y_pred _> y_meas_**	**y_pred _< y_meas_**
Quantitative (no threshold)	(y_pred_-y_meas_)^2^	(y_pred_-y_meas_)^2^
Upper boundary (threshold: greater)	0	(y_pred_-y_meas_)^2^
Lower boundary (threshold: lesser)	(y_pred_-y_meas_)^2^	0
