# Supplementary material for: Generating quantitative models describing the sequence specificity of biological processes with the stabilized matrix method
Source: BMC Bioinformatics. 2005 May 31;6:132. doi: 10.1186/1471-2105-6-132 (PMC1173087; doi:10.1186/1471-2105-6-132)
Supplement: Additional File 1 — A .zip file containing the SMM source code, documentation and examples for windows systems. [file 1471-2105-6-132-S1.zip › smmwin/readme.htm]

SMM


# SMM

The smm program implements the stabilized matrix method, which generates
quantitative models describing the sequence specificity of biological processe.
In essence, it uses sets of sequences with measured values to generate a tool
predicting experimental outcomes for new sequences. A detailed description is
provided in this manuscript.

This file should contain enough information to start using the smm program.
Refer to the smm project homepage for
updates. If you encounter any problems, please write an email to 
Bjoern Peters, who wrote the program and likes to talk about himself in
the third person.

## Contents of this file

- Overview of the distributed files
- Using the general XML Interface
- Using the simplified interface
- Compiling the source code
- License

## Files

Files contained in the smmwin or smmlinux archives

| Name | Description | Windows specific1 |
| /readme.htm | This readme file | No |
| /smm.exe | windows executable | Yes |
| /smm.xsd | schema file for xml input and output | No |
| /input/ | examples for input files | No |
| /output/ | examples for output files | No |
| /code/ \*.cpp, \*.h | C++ source code | No |
| /code/smm.ncb | Visual C++ .net project files | Yes |
| /code/smm.sln | Visual C++ .net project files | Yes |
| /code/smm.vcproj | Visual C++ .net project files | Yes |
| /code/Makefile | Makefile, tested for g++ 1.5 and higher | No |

1 Windows specific files are only contained in the smmwin archive

## The XML interface

This interface provides the most general and flexible way for using the smm
programm, as it allows to modify all internal parameters. This comes at a
price: the content of the input files may seem complicated, especially for
first time XML users. Therefore, a complementary interface designed for the
most common applications is also provided and described below.

For the XML interface, the general calling convention is

```
smm.exe [XMLFileName]
```

The specified file has to contain XML data from which the smm program extracts
all input parameters. In the examples below, it is assumed that the input files
are in the same directory as smm.exe. If this is not the case, path information
has to be added to the input file names.

### Training

The "input" directory contains three files with sample xml training data:

- TrainInput1.xml - contains peptides of length 4 with associated measurements
- TrainInput2.xml - contains peptides and a scoring matrix derived from a
  combinatorial peptide library
- TrainInput3.xml - contains peptides and a scoring matrix and specifies how the
  smm method should calculate the prediction tool

To run the examples, call the smm.exe file from the command line and give the
name of the input file as a parameter, e.g.:

```
smm.exe TrainInput1.xml
```

Note that the training process for the example files will take about one minute
on a 2 Ghz Pentium 4 PC, and no output is generated before the end of that
process.

In the first two examples, the smm method decides how to calculate the
prediction tool on the basis of the training set size. As enough training data
is present, a scoring matrix and pair coefficients are calculated. In the third
example, the <MatrixCalculation> element at the end of the input file
defines that no pair coefficients will be calculated (as no
<PairCalculation> element is present). The
<AdjustOffsetToSequenceData> element specifies that the offset of the
scoring matrix is adjusted to the peptide data, which is recommended if both
come from different experimental settings.

### Prediction

The three above examples generate output files at the location specified in the
TrainInput files. Each output file has a <SMMPredictor> element
containing a scoring matrix and (in the first two examples) pair coefficients.
To make a prediction, an <SMMPredictor> element along with sequences for
which predictions are to be made are passed to the smm.exe file. An example is
given in the PredictInput.xml file. To make a prediction, call

```
smm.exe PredictInput.xml
```

### Example output

For all the training and prediction examples mentioned in the previous section,
the output files that should be generated are contained in the /output/
directory. The exact coefficient values in the output files can vary between
systems, if their random number generator implementations (used during
cross-validation) differ.

### XML Schema

The smm.xsd file contains an xml schema defining what elements are allowed in
the training and prediction input files. Each element is annotated, to document
its intended use.

The smm.exe program expects the input files to be valid according to this
schema. If an undefined error occurs when running smm.exe, first make sure that
the input files validate against the schema. This is easy to do with freely
available software such as xmlspy, which
also comes with a nice schema viewer.

## Using the simplified interface

This is a limited interface, designed to provide easy access to the most common
SMM applications. It can only handle amino acid sequence data, and always uses
default parameters during the training process. By avoiding the use of XML, it
should be easy for any user to construct their own input files.

Following is a list of examples using this interface. They assume that the input files are in the same directory as smm.exe. If this
is not the case, path information has to be added to the input file names.

### Generate a scoring matrix

Enter at the command line:

```
smm.exe -tm TrainInput1.txt
```

The newly generated scoring matrix will in this case be saved as
'mat-TrainInput1.txt'. To save it under a different file name, e.g. 'mymatrix',
add a second file name:

```
smm.exe -tm TrainInput1.txt mymatrix
```

Choosing such a user defined filename for the output is always possible, but is
not repeated explicitly in the examples below.

### Generate a scoring matrix and pair coefficients

```
smm.exe -tp TrainInput1.txt
```

or

```
smm.exe -t TrainInput1.txt
```

The lower option only generates pair coefficients if the number of data points
in the training input is sufficient.

### Make a prediction

```
smm.exe -p mat-TrainInput1.txt sequences.txt
```

The first file specified has to contain a previously generated matrix with or
without pair coefficients. The second file contains the sequences for which a
prediction is supposed to be made. The output is saved as 'pred-sequences.txt'.

## Compiling the source code

The source code has been compiled and tested in Visual C++.Net on Windows XP,
and using g++ on Debian and Suse Linux distributions. In each case, the 
Gnu Scientific Library (GSL) has to be installed first. A 
GSL version for windows is available here. The only other non-standard
library used is TinyXml,
which is included in the source code.

The easiest way to compile under windows is to use the supplied Visual C++
project files. They assume a standard installation of the GSL libray at
C:\Program Files\GnuWin32.

To compile using g++, use the supplied Makefile. g++ has to be at least version
1.5 or higher.

## License Issues

The smm code itself is released under the 
zlib license, which is repeated below. Note that if you want to use
parts of the smm code relying on the GSL library, its 
GNU GPL license applies, which is somewhat restrictive regarding
commercial use.

### License for smm code (zlib)

This software is provided 'as-is', without any express or implied warranty. In
no event will the authors be held liable for any damages arising from the use
of this software. Permission is granted to anyone to use this software for any
purpose, including commercial applications, and to alter it and redistribute it
freely, subject to the following restrictions:

1. The origin of this software must not be misrepresented; you must not claim that
   you wrote the original software. If you use this software in a product, an
   acknowledgment in the product documentation would be appreciated but is not
   required.
2. Altered source versions must be plainly marked as such, and must not be
   misrepresented as being the original software.
3. This notice may not be removed or altered from any source distribution.
